# Protein-First, but Not Protein-Only: Rethinking Neurodegenerative Diseases Through Transgenic Mouse Models

**DOI:** 10.3390/neurolint18070139

**Published:** 2026-07-21

**Authors:** Chih-Wei Zeng

**Affiliations:** Department of Neuroscience, University of Texas Southwestern Medical Center, Dallas, TX 75390, USA; chih-wei.zeng@utsouthwestern.edu

**Keywords:** neurodegenerative diseases, transgenic mouse models, proteinopathy, Alzheimer’s disease, Parkinson’s disease, amyotrophic lateral sclerosis, tau, α-synuclein, TDP-43, neuroinflammation

## Abstract

Neurodegenerative diseases represent a major and growing global health burden. Although these disorders are often clinically defined by symptoms and affected brain regions, many are mechanistically linked to abnormal protein accumulation, misfolding, impaired proteostasis, RNA dysregulation, mitochondrial dysfunction, and neuroinflammation. In this Perspective article, I discuss major neurodegenerative diseases, including Alzheimer’s disease, Parkinson’s disease, dementia with Lewy bodies, multiple system atrophy, amyotrophic lateral sclerosis, frontotemporal dementia, Huntington’s disease, prion diseases, spinocerebellar ataxias, and spinal muscular atrophy, through the lens of disease-associated proteins and experimental modeling. I argue that a protein-centered framework provides a useful approach for understanding disease mechanisms and selecting transgenic mouse models, while recognizing that aging, cellular context, neuroinflammation, mitochondrial dysfunction, vascular dysfunction, and other disease modifiers also shape neurodegeneration. Transgenic and genetically engineered mouse models have been essential for dissecting the pathogenic roles of amyloid-β, tau, α-synuclein, TDP-43, SOD1, FUS, *C9ORF72*-associated dipeptide repeat proteins, mutant huntingtin, prion protein, ataxins, and SMN deficiency. However, these models have important limitations, including artificial overexpression, familial mutation bias, species differences, and incomplete representation of aging-related sporadic diseases. Rather than seeking a single “best” model, a more productive strategy is to adopt model portfolios tailored to specific biological questions and to integrate mouse studies with human cellular models, postmortem tissue, omics approaches, and biomarker-based validation. Such an approach may improve mechanistic insight, strengthen translational relevance, and enhance the predictive value of preclinical neurodegenerative disease research.

## 1. Introduction

**Figure 1:**
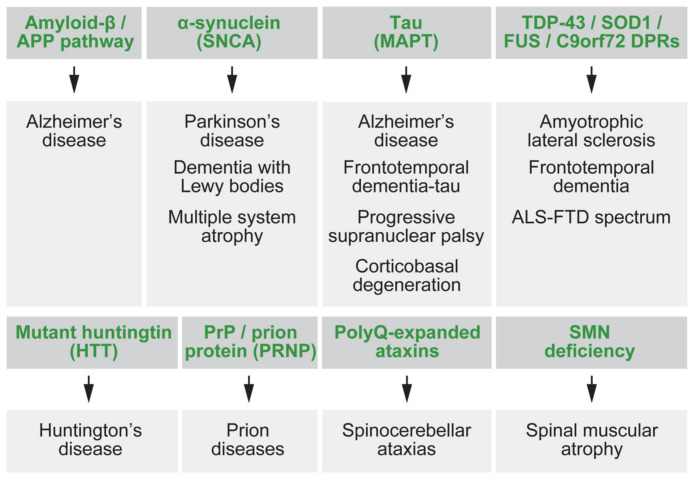
Major disease-associated proteins and pathogenic factors in neurodegeneration. This schematic summarizes key proteins and pathogenic factors linked to major neurodegenerative diseases. The map is intended as a protein-centered framework rather than a strict one-protein-one-disease classification, because several diseases share overlapping molecular mechanisms. Cellular context and additional disease modifiers, including aging, neuroinflammation, mitochondrial dysfunction, and vascular dysfunction, are further addressed in Figure 2 and Figure 3.

Major neurodegenerative diseases include Alzheimer’s disease (AD), Parkinson’s disease (PD), dementia with Lewy bodies, multiple system atrophy, amyotrophic lateral sclerosis (ALS), frontotemporal dementia (FTD), Huntington’s disease (HD), prion diseases, spinocerebellar ataxias, and spinal muscular atrophy (SMA). These disorders are characterized by progressive dysfunction and loss of selectively vulnerable neuronal populations and can be classified by clinical syndrome, anatomical pattern, genetic cause, or molecular pathology [1]. The burden of these disorders is increasing worldwide. The Global Burden of Disease 2019 Dementia Forecasting Collaborators estimated that 57.4 million people had dementia in 2019 and projected this number to increase to 152.8 million by 2050 [2]. PD is also rising globally, with the Global Burden of Disease Study estimating 6.1 million individuals living with PD in 2016, compared with 2.5 million in 1990 [3]. Although neurodegenerative diseases differ in clinical presentation, age of onset, anatomical vulnerability, and disease duration, many share common biological features, including abnormal protein aggregation, impaired proteostasis, mitochondrial dysfunction, synaptic failure, RNA dysregulation, glial activation, neuroinflammation, and selective neuronal vulnerability [4,5]. These shared mechanisms allow the organization of neurodegenerative diseases beyond symptom-based classification. In my opinion, a protein-centered framework provides a useful way to organize these diseases, including amyloid-β and tau in AD, α-synuclein (αSyn) in synucleinopathies, TDP-43-related mechanisms in ALS-FTD, mutant huntingtin in HD, PrP in prion diseases, expanded ataxins in spinocerebellar ataxias, and SMN deficiency in SMA [1,4] (Figure 1). Although disease-associated proteins provide powerful entry points into disease mechanisms, they do not fully explain cell-type vulnerability, aging dependence, regional selectivity, glial contribution, vascular influence, systemic metabolism, or therapeutic failure. In this Perspective, the protein-centered framework is therefore used as a practical organizing principle rather than as an exclusive explanation of neurodegeneration. Transgenic mouse models should accordingly be viewed as mechanistic instruments rather than complete replicas of human disease.

## 2. A Protein-Centered View of Major Neurodegenerative Diseases

A major strength of the proteinopathy concept is that it provides a molecular language across clinically diverse diseases. AD is pathologically associated with extracellular amyloid-β plaques and intracellular neurofibrillary tangles composed of hyperphosphorylated tau [6]. PD and dementia with Lewy bodies are strongly associated with neuronal Lewy bodies and Lewy neurites containing αSyn [7]. The discovery that *SNCA* mutations can cause familial PD provided additional genetic evidence linking αSyn to Parkinsonian neurodegeneration [8]. Multiple system atrophy is another α-synucleinopathy, but it differs from PD and dementia with Lewy bodies because αSyn accumulates prominently in oligodendrocytes as glial cytoplasmic inclusions [9,10]. This distinction illustrates an important principle: the same pathogenic protein can produce different disease phenotypes depending on cell type and anatomical context (Table 1). Tau biology provides another example. Tau pathology is present in AD, but tau also drives primary tauopathies, such as frontotemporal dementia associated with *MAPT* mutation, progressive supranuclear palsy, and corticobasal degeneration. Progressive supranuclear palsy and corticobasal degeneration are commonly considered four-repeat tauopathies, whereas AD contains both three- and four-repeat tau isoforms [11]. Therefore, tau identity alone cannot define the disease; tau isoform, cellular distribution, anatomical spread, and accompanying pathology matter as well. ALS and FTD further challenge the single-disease framework. TDP-43 was identified as a major disease protein in ALS and frontotemporal lobar degeneration with ubiquitinated inclusions [12]. A *C9ORF72* hexanucleotide repeat expansion was later identified as a major genetic cause of both ALS and FTD, linking RNA toxicity, dipeptide repeat proteins, and TDP-43 pathology to the ALS-FTD spectrum [13,14]. In addition, HD and several spinocerebellar ataxias are linked to expanded polyglutamine proteins, including mutant huntingtin and ataxins [4]. Prion diseases are uniquely associated with the conversion of normal prion protein into pathogenic misfolded conformers [15]. In contrast, SMA differs from classical protein-aggregation disorders because it is primarily caused by deficiency of survival motor neuron protein due to *SMN1* loss and incomplete compensation by *SMN2* [16,17]. Thus, a protein-centered map is highly useful, but it must be interpreted as a framework rather than a one-protein-one-disease rule.

### Why a Protein-Centered Framework Remains Useful

Neurodegenerative disease phenotypes are shaped by biological processes beyond proteinopathy, including aging, impaired proteostasis, mitochondrial dysfunction, RNA dysregulation, neuroinflammation, vascular dysfunction, metabolic stress, and interactions between neurons and glia [4,5]. These processes may precede, amplify, or arise downstream of disease-associated protein abnormalities, and their relative contributions vary across diseases and disease stages. Thus, a protein-centered framework does not imply that proteins are universally more important than other pathogenic processes.

In this Perspective, disease-associated proteins are prioritized as an organizing anchor because they connect several levels of investigation. They define major neuropathological categories and are often genetically linked to disease [1], can be manipulated directly in experimental models [27], and provide measurable molecular readouts that can support target engagement and biomarker studies [5]. This combination makes them particularly useful for formulating mechanistic questions, comparing diseases, and selecting experimental models according to a defined research purpose [28]. Their use as the primary organizing anchor is therefore practical and mechanistic rather than exclusive.

**Figure 2:**
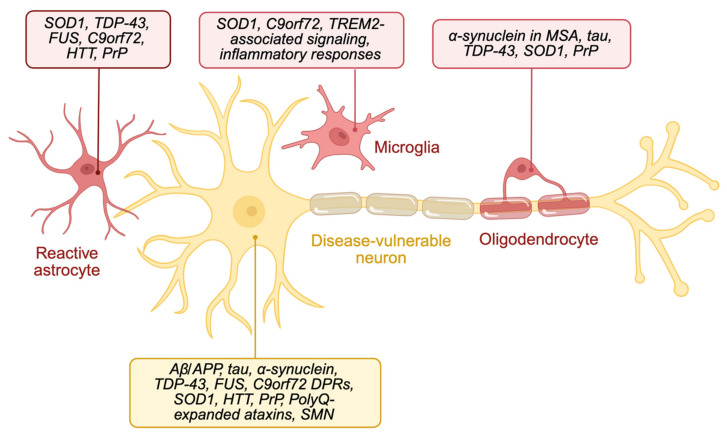
Cellular context of disease-associated proteins in neurodegeneration. Disease-associated proteins and related molecular responses occur across neurons, astrocytes, microglia, and oligodendrocytes. The same pathogenic protein can contribute to distinct phenotypes depending on cell type and anatomical context, as illustrated by neuronal α-synuclein pathology in Parkinson’s disease and dementia with Lewy bodies and oligodendroglial α-synuclein pathology in multiple system atrophy. The listed proteins and cellular responses are representative rather than exhaustive. Created in BioRender. Zeng, C. (2026) https://BioRender.com/pjvmc8l.

Cellular context further determines how protein-associated mechanisms are expressed. α-synuclein is mainly associated with neuronal inclusions in Parkinson’s disease and dementia with Lewy bodies [7], but it accumulates prominently in oligodendrocytes in multiple system atrophy [9,10]. TDP-43 pathology can predominate in motor or frontotemporal networks in amyotrophic lateral sclerosis and frontotemporal dementia [12], while glial responses can modify neuronal vulnerability and disease progression [5]. As illustrated in Figure 2, the proposed framework is protein-first because disease-associated proteins provide experimentally tractable anchors, but not protein-only because aging, cell type, inflammatory state, mitochondrial function, vascular health, and systemic biology influence whether and how neurodegeneration develops.

## 3. Transgenic Mouse Models as Mechanistic Tools

Transgenic and genetically engineered mouse models have been central to neurodegenerative disease research because they allow direct testing of disease-associated genes and proteins in vivo [27] (Table 2). These models can be used to study protein aggregation, synaptic dysfunction, neuronal loss, glial activation, behavioral impairment, disease progression, and therapeutic target engagement.

In AD research, the 5xFAD model overexpresses mutant human amyloid precursor protein (APP) and presenilin 1 (PSEN1) carrying five familial AD mutations and develops rapid amyloid deposition, neuroinflammation, synaptic dysfunction, and neuronal loss [29]. The 3xTg-AD model combines mutant APP, PSEN1, and tau and has been used to study interactions among amyloid-β accumulation, tau pathology, synaptic dysfunction, and memory impairment [21]. More recently, *App* knock-in models, including *App*^NL-F^ and *App*^NL-G-F^, were developed to avoid some limitations of APP overexpression while preserving amyloidogenic APP processing from the endogenous mouse *App* locus [30,31].

In PD and synucleinopathy research, A53T αSyn transgenic mice demonstrated that mutant human αSyn can drive neuronal α-synucleinopathy, movement disorder, and neurodegeneration [18]. Thy1-αSyn mice have also been widely used to examine progressive αSyn-associated motor and non-motor phenotypes [19]. For multiple system atrophy, oligodendrocyte-directed αSyn models, such as PLP-αSyn mice, have been used to study glial cytoplasmic inclusion-like pathology and neuron-glia interactions [20].

In ALS research, SOD1-G93A mice remain one of the most widely used models of motor neuron degeneration [33]. However, because most ALS cases are not caused by SOD1 mutations, TDP-43, FUS, and *C9ORF72* models are essential for modeling broader ALS-FTD mechanisms. TDP-43 A315T mice develop motor dysfunction and features relevant to ALS and frontotemporal lobar degeneration [24]. Inducible rNLS8 mice expressing human TDP-43 lacking a nuclear localization signal have been useful for studying the reversible and irreversible consequences of cytoplasmic TDP-43 accumulation [25]. In addition, *C9ORF72* BAC transgenic mice have been used to model repeat RNA foci, dipeptide repeat proteins, neurodegeneration, and ALS-FTD-related phenotypes [26].

Tauopathy models have also been highly influential. rTg4510 mice expressing P301L mutant tau showed that suppression of mutant tau could improve memory function, even when neurofibrillary pathology was already present [23]. PS19 mice expressing P301S mutant human tau develop tau aggregation, synaptic loss, microglial activation, and neurodegeneration, making them useful for studying tau-mediated disease mechanisms [22].

In HD, R6/2 mice expressing exon 1 of mutant human huntingtin (HTT) with an expanded CAG repeat develop a rapid and severe neurological phenotype [36]. Full-length models, such as YAC128 and BACHD, as well as knock-in models such as zQ175, provide complementary systems for studying mutant huntingtin in a more genetically faithful context [37,38,39].

Prion mouse models have been particularly powerful because they allow direct testing of infectivity, species barriers, and the requirement for host prion protein expression. *Prnp* knockout mice are resistant to scrapie, demonstrating that host prion protein is required for disease pathogenesis [40]. PrP-overexpressing Tga20 mice and humanized *PRNP* mice further enabled analysis of prion strain behavior and species barriers [41,42].

Spinocerebellar ataxia models have shown how polyglutamine-expanded ataxins can result in selective neuronal dysfunction. *ATXN1*[82Q] transgenic mice provided an early model of SCA1-like cerebellar degeneration [43]. YAC transgenic mice carrying pathological Machado-Joseph disease/SCA3 alleles developed progressive cerebellar phenotypes [44], while SCA7 mouse models demonstrated that polyglutamine-expanded ataxin-7 can disrupt retinal transcriptional programs and cause cone-rod dystrophy [45].

SMA mouse models have been particularly important for therapeutic development. *Smn*-deficient mice rescued with human *SMN2* transgenes reproduce key features of SMA and demonstrate the dosage-dependent importance of survival motor neuron (SMN) protein [16,17]. SMNΔ7 mice have also been widely used to study SMA pathogenesis and preclinical therapeutic strategies [46].

## 4. Strengths of Transgenic Mouse Models

The strongest advantage of transgenic mouse models is their ability to test causality. Human postmortem studies can identify protein aggregates and neuronal loss, but they often cannot determine whether these changes are causes, consequences, or late-stage markers. In contrast, mouse models allow direct manipulation of genes, proteins, cell types, and disease stages [27]. Mouse models also enable longitudinal studies of disease progression. Researchers can examine early molecular changes before overt neuronal loss, follow behavioral and pathological changes over time, and test therapeutic interventions at defined disease stages. This is particularly important because human neurodegenerative diseases often begin years or decades before clinical diagnosis [27,28]. Another strength is the ability to study interactions between neurons and non-neuronal cells. Microglia, astrocytes, oligodendrocytes, endothelial cells, and peripheral immune cells all influence neurodegenerative disease progression [5]. For example, amyloid and tau models can be used to study neuroinflammation, synaptic dysfunction, and glial responses in AD-related contexts. αSyn models can be used to examine neuron-glia interactions in synucleinopathies. ALS models can help define how astrocytes, microglia, and peripheral neuromuscular components contribute to motor neuron degeneration. Transgenic mice are also essential for preclinical evaluation. They allow testing of target engagement, pharmacodynamics, biomarker responses, treatment timing, dosing, toxicity, and tissue distribution. Even when a mouse model does not fully predict human clinical efficacy, it can still determine whether a therapeutic strategy produces the intended biological effect in vivo [28].

## 5. Limitations of Current Mouse Models

Despite their value, transgenic mouse models have important limitations. First, many widely used models rely on artificial overexpression of mutant human proteins. This approach can accelerate pathology and generate robust phenotypes, but it can also produce non-physiological effects. In AD research, APP-overexpressing models may rapidly generate amyloid pathology, but they can also overproduce APP fragments and related metabolites that may complicate the interpretation of amyloid-β-specific mechanisms [31]. Second, many models are based on rare inherited mutations, whereas most human neurodegenerative diseases are sporadic and age-associated. Familial *APP*, *PSEN1*, and *PSEN2* mutations have been highly informative for AD research, but they do not fully represent late-onset sporadic AD. Similarly, SOD1-G93A mice are powerful and reproducible ALS models, but SOD1 mutations account for only a subset of human ALS cases. Tau mutation models are highly useful for frontotemporal dementia-tau, but diseases such as progressive supranuclear palsy and corticobasal degeneration are usually sporadic four-repeat tauopathies [11]. Third, mice do not naturally reproduce the full complexity of human brain aging. Human neurodegenerative diseases develop over decades and are shaped by aging, vascular dysfunction, metabolic disease, environmental exposure, immune history, and genetic risk. Mouse models compress disease progression into months or a few years, which can limit their ability to represent the chronic biological environment in which human neurodegeneration emerges [27,47]. Species differences also matter. Mouse and human neurons differ in size, connectivity, lifespan, gene regulation, and vulnerability. Human astrocytes, microglia, and oligodendrocytes also differ from their mouse counterparts. These differences may partly explain why therapeutic success in mouse models often fails to translate into clinical benefit [28,47]. For these reasons, transgenic mice should not be considered miniature patients. Instead, they are best viewed as mechanistic instruments. They are powerful tools for addressing specific biological questions, but they should not be expected to reproduce every feature of human disease.

## 6. From Single Models to Model Portfolios

**Figure 3:**
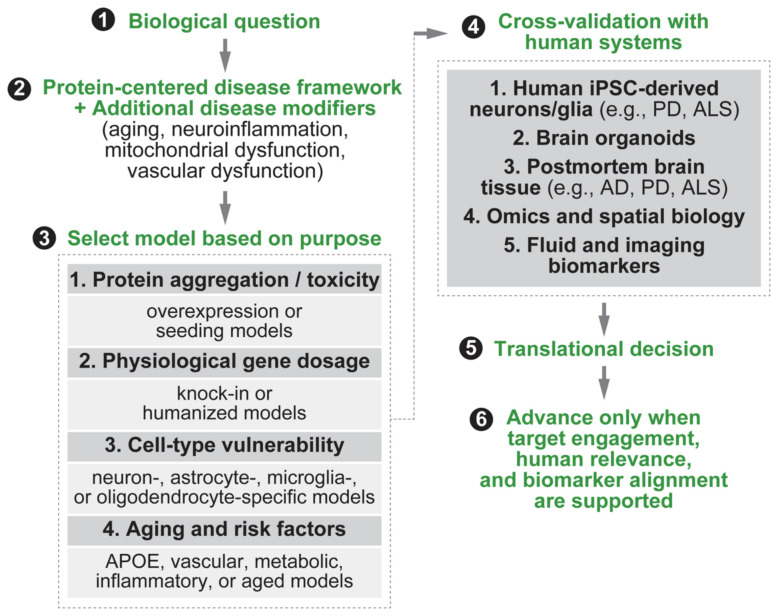
Model-portfolio strategy for neurodegenerative disease research. The model-selection process begins with a defined biological question and integrates a protein-centered framework with additional disease modifiers, including aging, neuroinflammation, mitochondrial dysfunction, and vascular dysfunction. Models are then selected according to experimental purpose and cross-validated using human iPSC-derived neurons and glia, brain organoids, postmortem tissue, omics and spatial biology, and fluid or imaging biomarkers before informing translational decisions.

A major problem in neurodegenerative disease research is the tendency to ask which mouse model is “best.” In my view, this is the wrong question. A better question is: *Which model is appropriate for the specific biological question being asked?* This principle has been emphasized in discussions of translational animal modeling, where model choice should be guided by experimental purpose rather than disease name alone [28]. For AD, no single mouse model can represent the entire disorder. 5xFAD and related amyloid models are useful for studying amyloid deposition and amyloid-associated inflammation [29]. Tau models such as PS19 and rTg4510 are more appropriate for investigating tau aggregation and tau-mediated neurodegeneration [22,23]. App knock-in models are useful when physiological APP expression is important [30]. In addition, *APOE*-targeted, vascular, metabolic, inflammatory, and aging-related models may be required to address late-onset sporadic AD more directly. For PD, αSyn transgenic models are appropriate for studying αSyn aggregation and αSyn-associated toxicity, whereas *LRRK2*, *PINK1*, *Parkin*, *GBA*, mitochondrial, and lysosomal models may be more relevant for addressing specific genetic or cellular mechanisms. For multiple system atrophy, oligodendrocyte-directed αSyn models are more appropriate than purely neuronal αSyn models because oligodendroglial pathology is a defining feature of the human disease [9,10,20]. For ALS and FTD, SOD1, TDP-43, FUS, *C9ORF72*, and progranulin models each address different aspects of the disease spectrum. A therapy designed to reduce SOD1 toxicity should be tested in SOD1-relevant systems, whereas a therapy targeting TDP-43 mislocalization should be evaluated in TDP-43-based models. Similarly, *C9ORF72*-targeted therapies require models that reproduce repeat RNA, dipeptide repeat proteins, and downstream neurodegenerative phenotypes [26]. Thus, a model-portfolio approach is more scientifically useful than relying on a single “best” model. Different models should be selected according to the specific biological question being addressed, whether the goal is to study disease initiation, protein aggregation, cell-type vulnerability, glial response, circuit dysfunction, biomarker dynamics, or therapeutic target engagement. Figure 3 summarizes this model-selection process by integrating a protein-centered framework with additional disease modifiers before model selection and subsequent cross-validation using human-based systems.

## 7. Integrating Mouse Models with Human-Based Systems

The future of neurodegenerative disease research should not abandon mouse models. Instead, mouse models should be integrated with human-based systems. Human induced pluripotent stem cell (iPSC)-derived neurons, astrocytes, microglia, oligodendrocytes, brain organoids, postmortem tissue, single-cell omics, spatial transcriptomics, proteomics, and fluid biomarkers can provide information that mouse models alone cannot provide [5,48]. Patient-derived iPSC models retain donor-specific genetic backgrounds and can be differentiated into disease-relevant cell types [48,49]. In PD, *LRRK2* G2019S iPSC-derived dopaminergic neurons show increased oxidative-stress responses and elevated α-synuclein, whereas *SNCA*-triplication neurons accumulate α-synuclein and are susceptible to oxidative stress [50,51]. In amyotrophic lateral sclerosis, *C9ORF72*-expanded iPSC-derived motor neurons reproduce RNA foci and related molecular abnormalities and can be used to evaluate targeted interventions [52]. These systems are therefore useful for mechanistic studies and therapeutic testing. Postmortem tissue can validate whether experimental findings occur in relevant human brain regions and cell types. Such studies identified neuronal α-synuclein in Lewy bodies, oligodendroglial α-synuclein in multiple system atrophy, and TDP-43 pathology in amyotrophic lateral sclerosis and frontotemporal dementia [7,9,12]. However, because postmortem samples usually represent late-stage disease, they are more suitable for pathological validation than for determining temporal causality.

In my opinion, the goal is not to replace mice with human systems, but to connect them. Mouse models test causality in vivo, human cellular systems establish human genetic relevance, postmortem tissue validates pathology, and biomarkers link experimental mechanisms to patients. Therapeutic strategies should advance only when target engagement, human relevance, and biomarker alignment are supported.

## 8. Conclusions

Neurodegenerative diseases are often named according to clinical symptoms, but they are increasingly understood through molecular mechanisms. A protein-centered framework provides a useful way to organize major diseases, including AD, PD, dementia with Lewy bodies, multiple system atrophy, ALS, FTD, HD, prion diseases, spinocerebellar ataxias, and spinal muscular atrophy. The value of this framework lies in connecting neuropathology, genetics, experimental model design, biomarkers, and therapeutic targets, rather than in assuming that proteins are the only or universally dominant drivers of disease. However, neurodegeneration is not caused by proteins alone. Disease phenotype depends on protein identity, cell type, brain region, aging, glial response, immune signaling, genetic risk, vascular health, and systemic biology. Transgenic mouse models remain powerful tools for investigating disease mechanisms, but they should be viewed as mechanistic instruments rather than complete replicas of human disease. The field should move toward model portfolios, in which different mouse models are selected according to specific biological questions and subsequently validated using human cellular systems, postmortem tissue, omics approaches, and clinical biomarkers. Such an approach is more likely to improve mechanistic understanding, strengthen translational relevance, and enhance the predictive value of preclinical research. A mature view of neurodegenerative disease should retain the proteinopathy framework while expanding beyond it. Proteins may define the entry point into disease, but the full degenerative process is shaped by the cellular and systemic environment in which those proteins act.

## Figures and Tables

**Table 1:** Same protein, different disease context.

Protein	Disease Context	Associated Disease	Model Implication	References
α-synuclein	Neuronal inclusions	Parkinson’s disease; DLB	Use neuronal α-synuclein models	[7,18,19]
	Oligodendroglial inclusions	Multiple system atrophy	Use oligodendrocyte-directed α-synuclein models	[9,10,20]
Tau	Mixed 3R and 4R tau with amyloid-β	Alzheimer’s disease	Use amyloid, tau, or combined AD models	[6,21]
	Predominantly 4R tau	PSP; CBD	Use 4R tau or P301S/P301L tau models	[11,22]
	*MAPT* mutation	FTD-tau	Use mutation-driven tauopathy models	[22,23]
TDP-43	Motor neuron pathology	ALS	Use TDP-43 ALS models	[12,24,25]
	Frontotemporal cortical pathology	FTD-TDP	Use cortical and behavior-focused models	[12,25]
*C9ORF72*	Repeat RNA, DPRs, TDP-43 pathology	ALS-FTD spectrum	Use *C9ORF72* repeat models	[13,14,26]

**Table 2:** Major neurodegenerative diseases and representative mouse models.

Disease	Major Protein or Pathway	Representative Mouse Models	Main Use	Key Limitation	References
Alzheimer’s disease	amyloid-β, APP, PSEN1/PSEN2, tau	5xFAD; APP/PS1; 3xTg-AD; *App*^NL-F^; *App*^NL-G-F^	Amyloid pathology; amyloid-tau interaction; neuroinflammation	Familial mutation bias; overexpression artifacts in some models	[21,29,30,31]
Parkinson’s disease/DLB	α-synuclein; LRRK2	A53T α-synuclein; Thy1-α-synuclein; LRRK2-G2019S	Synucleinopathy; motor phenotypes; dopaminergic vulnerability	Incomplete Lewy body and nigral degeneration phenotypes	[18,19,32]
Multiple system atrophy	Oligodendroglial α-synuclein	PLP-α-synuclein; MBP-α-synuclein; CNP-α-synuclein	Glial cytoplasmic inclusions; neuron-glia interaction	Forced glial α-synuclein expression	[9,10,20]
Amyotrophic lateral sclerosis	SOD1; TDP-43; FUS; *C9ORF72*-associated DPRs	SOD1-G93A; TDP-43 A315T; rNLS8; *C9ORF72* BAC; FUS-R521C	Motor neuron degeneration; NMJ loss; RNA toxicity	Model-specific phenotypes; SOD1 represents a subset	[24,25,26,33,34]
Frontotemporal dementia	Tau; TDP-43; FUS; *GRN*; *C9ORF72*	rTg4510; PS19; *Grn*-deficient mice; *C9ORF72* BAC	Cortical degeneration; behavioral phenotypes; tau/TDP-43 biology	Highly heterogeneous disease mechanisms	[22,23,26,35]
PSP/CBD	Predominantly 4R tau	PS19 (P301S tau); rTg4510 (P301L tau)	Tau aggregation; motor dysfunction	Limited modeling of sporadic glial tauopathy	[11,22,23]
Huntington’s disease	Mutant huntingtin	R6/2; YAC128; BACHD; zQ175	PolyQ toxicity; striatal dysfunction; HTT-lowering studies	R6/2 is rapid and fragment-based; knock-ins are slower	[36,37,38,39]
Prion diseases	Misfolded PrP/*PRNP*	*Prnp* knockout; Tga20; humanized *PRNP* mice	Infectivity; strain biology; species barrier	Biosafety and strain specificity	[40,41,42]
Spinocerebellar ataxias	PolyQ-expanded ataxins	*ATXN1*[82Q]; SCA3-YAC; SCA7 models	Purkinje cell dysfunction; cerebellar degeneration	Subtype-specific mechanisms	[43,44,45]
Spinal muscular atrophy	SMN deficiency	*SMN2*-rescue mice; SMNΔ7; Taiwanese SMA model	SMN dosage; motor unit pathology; therapy testing	Neonatal severity; developmental component	[16,17,46]

## Data Availability

No new data were created or analyzed in this study. Data sharing is not applicable to this article.

## Abbreviations

3R tau	tau isoforms containing three microtubule-binding repeats
3xTg-AD	triple-transgenic Alzheimer’s disease model
4R tau	tau isoforms containing four microtubule-binding repeats
5xFAD	mouse model carrying five familial Alzheimer’s disease mutations
Aβ	amyloid-β
αSyn	α-synuclein
AD	Alzheimer’s disease
ALS	amyotrophic lateral sclerosis
ALS-FTD	amyotrophic lateral sclerosis-frontotemporal dementia spectrum
APOE	apolipoprotein E
APP	amyloid precursor protein
ATXN1	ataxin 1
BAC	bacterial artificial chromosome
BACHD	bacterial artificial chromosome Huntington’s disease model
*C9ORF72*	chromosome 9 open reading frame 72
CAG	cytosine-adenine-guanine trinucleotide repeat
CBD	corticobasal degeneration
CNP	2′,3′-cyclic nucleotide 3′-phosphodiesterase
DLB	dementia with Lewy bodies
DPRs	dipeptide repeat proteins
FTD	frontotemporal dementia
FTD-tau	frontotemporal dementia with tau pathology
FTD-TDP	frontotemporal dementia with TDP-43 proteinopathy
FUS	fused in sarcoma
GBA	glucocerebrosidase
GRN	progranulin gene
HD	Huntington’s disease
HTT	huntingtin
iPSC	induced pluripotent stem cell
LRRK2	leucine-rich repeat kinase 2
MAPT	microtubule-associated protein tau
MBP	myelin basic protein
MSA	multiple system atrophy
NMJ	neuromuscular junction
PD	Parkinson’s disease
PINK1	PTEN-induced kinase 1
PLP	proteolipid protein
PolyQ	polyglutamine
PrP	prion protein
PRNP	prion protein gene
PSEN1	presenilin 1
PSEN2	presenilin 2
PSP	progressive supranuclear palsy
RNA	ribonucleic acid
SCA	spinocerebellar ataxia
SMA	spinal muscular atrophy
SMN	survival motor neuron
SMN1	survival motor neuron 1
SMN2	survival motor neuron 2
SNCA	α-synuclein gene
SOD1	superoxide dismutase 1
TDP-43	TAR DNA-binding protein 43
TREM2	triggering receptor expressed on myeloid cells 2
YAC	yeast artificial chromosome

## References

[1] Dugger B.N., Dickson D.W. (2017). Pathology of Neurodegenerative Diseases. Cold Spring Harb. Perspect. Biol..

[2] GBD 2019 Dementia Forecasting Collaborators (2022). Estimation of the global prevalence of dementia in 2019 and forecasted prevalence in 2050: An analysis for the Global Burden of Disease Study 2019. Lancet Public Health.

[3] GBD 2016 Parkinson’s Disease Collaborators (2018). Global, regional, and national burden of Parkinson’s disease, 1990–2016: A systematic analysis for the Global Burden of Disease Study 2016. Lancet Neurol..

[4] Ross C.A., Poirier M.A. (2004). Protein aggregation and neurodegenerative disease. Nat. Med..

[5] Wilson D.M., Cookson M.R., Van Den Bosch L., Zetterberg H., Holtzman D.M., Dewachter I. (2023). Hallmarks of neurodegenerative diseases. Cell.

[6] Selkoe D.J., Hardy J. (2016). The amyloid hypothesis of Alzheimer’s disease at 25 years. EMBO Mol. Med..

[7] Spillantini M.G., Schmidt M.L., Lee V.M., Trojanowski J.Q., Jakes R., Goedert M. (1997). Alpha-synuclein in Lewy bodies. Nature.

[8] Polymeropoulos M.H., Lavedan C., Leroy E., Ide S.E., Dehejia A., Dutra A., Pike B., Root H., Rubenstein J., Boyer R. (1997). Mutation in the alpha-synuclein gene identified in families with Parkinson’s disease. Science.

[9] Tu P.H., Galvin J.E., Baba M., Giasson B., Tomita T., Leight S., Nakajo S., Iwatsubo T., Trojanowski J.Q., Lee V.M. (1998). Glial cytoplasmic inclusions in white matter oligodendrocytes of multiple system atrophy brains contain insoluble alpha-synuclein. Ann. Neurol..

[10] Lee H.-J., Ricarte D., Ortiz D., Lee S.-J. (2019). Models of multiple system atrophy. Exp. Mol. Med..

[11] Rösler T.W., Tayaranian Marvian A., Brendel M., Nykänen N.P., Höllerhage M., Schwarz S.C., Hopfner F., Koeglsperger T., Respondek G., Schweyer K. (2019). Four-repeat tauopathies. Prog. Neurobiol..

[12] Neumann M., Sampathu D.M., Kwong L.K., Truax A.C., Micsenyi M.C., Chou T.T., Bruce J., Schuck T., Grossman M., Clark C.M. (2006). Ubiquitinated TDP-43 in frontotemporal lobar degeneration and amyotrophic lateral sclerosis. Science.

[13] DeJesus-Hernandez M., Mackenzie I.R., Boeve B.F., Boxer A.L., Baker M., Rutherford N.J., Nicholson A.M., Finch N.A., Flynn H., Adamson J. (2011). Expanded GGGGCC Hexanucleotide Repeat in Noncoding Region of C9ORF72 Causes Chromosome 9p-Linked FTD and ALS. Neuron.

[14] Renton A.E., Majounie E., Waite A., Simón-Sánchez J., Rollinson S., Gibbs J.R., Schymick J.C., Laaksovirta H., van Swieten J.C., Myllykangas L. (2011). A hexanucleotide repeat expansion in C9ORF72 is the cause of chromosome 9p21-linked ALS-FTD. Neuron.

[15] Prusiner S.B. (1998). Prions. Proc. Natl. Acad. Sci. USA.

[16] Monani U.R., Sendtner M., Coovert D.D., Parsons D.W., Andreassi C., Le T.T., Jablonka S., Schrank B., Rossoll W., Prior T.W. (2000). The human centromeric survival motor neuron gene (SMN2) rescues embryonic lethality in Smn(−/−) mice and results in a mouse with spinal muscular atrophy. Hum. Mol. Genet..

[17] Hsieh-Li H.M., Chang J.G., Jong Y.J., Wu M.H., Wang N.M., Tsai C.H., Li H. (2000). A mouse model for spinal muscular atrophy. Nat. Genet..

[18] Giasson B.I., Duda J.E., Quinn S.M., Zhang B., Trojanowski J.Q., Lee V.M.-Y. (2002). Neuronal α-Synucleinopathy with Severe Movement Disorder in Mice Expressing A53T Human α-Synuclein. Neuron.

[19] Chesselet M.-F., Richter F., Zhu C., Magen I., Watson M.B., Subramaniam S.R. (2012). A Progressive Mouse Model of Parkinson’s Disease: The Thy1-aSyn (‘Line 61’) Mice. Neurotherapeutics.

[20] Yazawa I., Giasson B.I., Sasaki R., Zhang B., Joyce S., Uryu K., Trojanowski J.Q., Lee V.M. (2005). Mouse model of multiple system atrophy alpha-synuclein expression in oligodendrocytes causes glial and neuronal degeneration. Neuron.

[21] Oddo S., Caccamo A., Shepherd J.D., Murphy M.P., Golde T.E., Kayed R., Metherate R., Mattson M.P., Akbari Y., LaFerla F.M. (2003). Triple-transgenic model of Alzheimer’s disease with plaques and tangles: Intracellular Abeta and synaptic dysfunction. Neuron.

[22] Yoshiyama Y., Higuchi M., Zhang B., Huang S.M., Iwata N., Saido T.C., Maeda J., Suhara T., Trojanowski J.Q., Lee V.M. (2007). Synapse loss and microglial activation precede tangles in a P301S tauopathy mouse model. Neuron.

[23] Santacruz K., Lewis J., Spires T., Paulson J., Kotilinek L., Ingelsson M., Guimaraes A., DeTure M., Ramsden M., McGowan E. (2005). Tau suppression in a neurodegenerative mouse model improves memory function. Science.

[24] Wegorzewska I., Bell S., Cairns N.J., Miller T.M., Baloh R.H. (2009). TDP-43 mutant transgenic mice develop features of ALS and frontotemporal lobar degeneration. Proc. Natl. Acad. Sci. USA.

[25] Walker A.K., Spiller K.J., Ge G., Zheng A., Xu Y., Zhou M., Tripathy K., Kwong L.K., Trojanowski J.Q., Lee V.M. (2015). Functional recovery in new mouse models of ALS/FTLD after clearance of pathological cytoplasmic TDP-43. Acta Neuropathol..

[26] Liu Y., Pattamatta A., Zu T., Reid T., Bardhi O., Borchelt D.R., Yachnis A.T., Ranum L.P. (2016). C9orf72 BAC Mouse Model with Motor Deficits and Neurodegenerative Features of ALS/FTD. Neuron.

[27] Dawson T.M., Golde T.E., Lagier-Tourenne C. (2018). Animal models of neurodegenerative diseases. Nat. Neurosci..

[28] Fisher E.M.C., Bannerman D.M. (2019). Mouse models of neurodegeneration: Know your question, know your mouse. Sci. Transl. Med..

[29] Oakley H., Cole S.L., Logan S., Maus E., Shao P., Craft J., Guillozet-Bongaarts A., Ohno M., Disterhoft J., Van Eldik L. (2006). Intraneuronal β-amyloid aggregates, neurodegeneration, and neuron loss in transgenic mice with five familial Alzheimer’s disease mutations: Potential factors in amyloid plaque formation. J. Neurosci..

[30] Saito T., Matsuba Y., Mihira N., Takano J., Nilsson P., Itohara S., Iwata N., Saido T.C. (2014). Single App knock-in mouse models of Alzheimer’s disease. Nat. Neurosci..

[31] Sasaguri H., Nilsson P., Hashimoto S., Nagata K., Saito T., De Strooper B., Hardy J., Vassar R., Winblad B., Saido T.C. (2017). APP mouse models for Alzheimer’s disease preclinical studies. EMBO J..

[32] Li X., Patel J.C., Wang J., Avshalumov M.V., Nicholson C., Buxbaum J.D., Elder G.A., Rice M.E., Yue Z. (2010). Enhanced striatal dopamine transmission and motor performance with LRRK2 overexpression in mice is eliminated by familial Parkinson’s disease mutation G2019S. J. Neurosci..

[33] Gurney M.E., Pu H., Chiu A.Y., Dal Canto M.C., Polchow C.Y., Alexander D.D., Caliendo J., Hentati A., Kwon Y.W., Deng H.X. (1994). Motor neuron degeneration in mice that express a human Cu, Zn superoxide dismutase mutation. Science.

[34] Qiu H., Lee S., Shang Y., Wang W.Y., Au K.F., Kamiya S., Barmada S.J., Finkbeiner S., Lui H., Carlton C.E. (2014). ALS-associated mutation FUS-R521C causes DNA damage and RNA splicing defects. J. Clin. Investig..

[35] Yin F., Banerjee R., Thomas B., Zhou P., Qian L., Jia T., Ma X., Ma Y., Iadecola C., Beal M.F. (2010). Exaggerated inflammation, impaired host defense, and neuropathology in progranulin-deficient mice. J. Exp. Med..

[36] Mangiarini L., Sathasivam K., Seller M., Cozens B., Harper A., Hetherington C., Lawton M., Trottier Y., Lehrach H., Davies S.W. (1996). Exon 1 of the HD gene with an expanded CAG repeat is sufficient to cause a progressive neurological phenotype in transgenic mice. Cell.

[37] Slow E.J., van Raamsdonk J., Rogers D., Coleman S.H., Graham R.K., Deng Y., Oh R., Bissada N., Hossain S.M., Yang Y.Z. (2003). Selective striatal neuronal loss in a YAC128 mouse model of Huntington disease. Hum. Mol. Genet..

[38] Gray M., Shirasaki D.I., Cepeda C., André V.M., Wilburn B., Lu X.H., Tao J., Yamazaki I., Li S.H., Sun Y.E. (2008). Full-Length Human Mutant Huntingtin with a Stable Polyglutamine Repeat Can Elicit Progressive and Selective Neuropathogenesis in BACHD Mice. J. Neurosci..

[39] Menalled L.B., Kudwa A.E., Miller S., Fitzpatrick J., Watson-Johnson J., Keating N., Ruiz M., Mushlin R., Alosio W., McConnell K. (2012). Comprehensive behavioral and molecular characterization of a new knock-in mouse model of Huntington’s disease: zQ175. PLoS ONE.

[40] Büeler H., Aguzzi A., Sailer A., Greiner R.A., Autenried P., Aguet M., Weissmann C. (1993). Mice devoid of PrP are resistant to scrapie. Cell.

[41] Fischer M., Rülicke T., Raeber A., Sailer A., Moser M., Oesch B., Brandner S., Aguzzi A., Weissmann C. (1996). Prion protein (PrP) with amino-proximal deletions restoring susceptibility of PrP knockout mice to scrapie. EMBO J..

[42] Collinge J., Palmer M.S., Sidle K.C., Hill A.F., Gowland I., Meads J., Asante E., Bradley R., Doey L.J., Lantos P.L. (1995). Unaltered susceptibility to BSE in transgenic mice expressing human prion protein. Nature.

[43] Burright E.N., Clark H.B., Servadio A., Matilla T., Feddersen R.M., Yunis W.S., Duvick L.A., Zoghbi H.Y., Orr H.T. (1995). SCA1 transgenic mice: A model for neurodegeneration caused by an expanded CAG trinucleotide repeat. Cell.

[44] Cemal C.K., Carroll C.J., Lawrence L., Lowrie M.B., Ruddle P., Al-Mahdawi S., King R.H., Pook M.A., Huxley C., Chamberlain S. (2002). YAC transgenic mice carrying pathological alleles of the MJD1 locus exhibit a mild and slowly progressive cerebellar deficit. Hum. Mol. Genet..

[45] La Spada A.R., Fu Y.H., Sopher B.L., Libby R.T., Wang X., Li L.Y., Einum D.D., Huang J., Possin D.E., Smith A.C. (2001). Polyglutamine-expanded ataxin-7 antagonizes CRX function and induces cone-rod dystrophy in a mouse model of SCA7. Neuron.

[46] Le T.T., Pham L.T., Butchbach M.E., Zhang H.L., Monani U.R., Coovert D.D., Gavrilina T.O., Xing L., Bassell G.J., Burghes A.H. (2005). SMNDelta7, the major product of the centromeric survival motor neuron (SMN2) gene, extends survival in mice with spinal muscular atrophy and associates with full-length SMN. Hum. Mol. Genet..

[47] Ransohoff R.M. (2018). All (animal) models (of neurodegeneration) are wrong. Are they also useful?. J. Exp. Med..

[48] Okano H., Morimoto S. (2022). iPSC-based disease modeling and drug discovery in cardinal neurodegenerative disorders. Cell Stem Cell.

[49] Zeng C.-W. (2025). Stem Cell-Based Approaches for Spinal Cord Injury: The Promise of iPSCs. Biology.

[50] Nguyen H.N., Byers B., Cord B., Shcheglovitov A., Byrne J., Gujar P., Kee K., Schüle B., Dolmetsch R.E., Langston W. (2011). LRRK2 mutant iPSC-derived DA neurons demonstrate increased susceptibility to oxidative stress. Cell Stem Cell.

[51] Byers B., Cord B., Nguyen H.N., Schüle B., Fenno L., Lee P.C., Deisseroth K., Langston J.W., Pera R.R., Palmer T.D. (2011). SNCA triplication Parkinson’s patient’s iPSC-derived DA neurons accumulate α-synuclein and are susceptible to oxidative stress. PLoS ONE.

[52] Sareen D., O’Rourke J.G., Meera P., Muhammad A.K., Grant S., Simpkinson M., Bell S., Carmona S., Ornelas L., Sahabian A. (2013). Targeting RNA foci in iPSC-derived motor neurons from ALS patients with a C9ORF72 repeat expansion. Sci. Transl. Med..

